# In Whom Do We Trust? A Multifoci Person-Centered Perspective on Institutional Trust during COVID-19

**DOI:** 10.3390/ijerph19031815

**Published:** 2022-02-05

**Authors:** Lixin Jiang, Erica L. Bettac, Hyun Jung Lee, Tahira M. Probst

**Affiliations:** 1 School of Psychology, University of Auckland, Auckland 1010, New Zealand; 2 Department of Psychology, Washington State University Vancouver, Vancouver, WA 98686, USA; erica.bettac@wsu.edu (E.L.B.); hyunjung.lee2@wsu.edu (H.J.L.); probst@wsu.edu (T.M.P.)

**Keywords:** institutional trust, latent profile analysis, trust in government, employee attitudes, COVID-19

## Abstract

Institutional trust plays a crucial role when a nation is facing mega crises (e.g., the COVID-19 pandemic) and has implications for employee work experiences and well-being. To date, researchers largely consider how institutional trust or trust in government may predict variables of interest in isolation. However, this variable-centered perspective ignores the possibility that there are subpopulations of employees who may differ in their trust in different institutions (i.e., the state government, the federal government). To address this, we examined institutional trust with two foci (i.e., trust in state government and trust in federal government) from a person-centered perspective. Using latent profile analysis and data from 492 U.S.-based employees, we identified five trust profiles: high trustors, federal trustors, state trustors, the ambivalent, and distrusters, and found that these profiles differentially predicted attitudes towards and behavioral compliance with CDC recommended COVID-19 prevention practices, job insecurity, affective commitment, helping behavior, and psychological well-being.

## 1. Introduction

In recent years, there has been an erosion of institutional trust in American government. In 2020, one in five American adults said that they trust the federal government to “do the right thing” just about always or most of the time, whereas four in five in 1964 said so [1]. Indeed, since 2007, public trust in federal government has generally fluctuated between 20% and 25% [1].

However, institutional trust, or beliefs about the degree to which the government is honest, cares for the public, has benevolent and caring intentions towards the public, and makes an effort in good faith to react to the needs and concerns of the public [2], is important, especially when a nation faces enormous crises such as the COVID-19 pandemic [3,4] because institutional trust is vital for the operation of democracy, efficient governance, institutional productivity, societal interactions, and compliance with governmental decisions [5,6,7,8].

Indeed, research has repeatedly shown that trust is a key determinant in the public’s compliance with regulations during a pandemic [9,10,11,12]. In the current COVID-19 pandemic, leaders of countries that rank high on interpersonal trust in the World Values survey were able to react to the pandemic quickly, put forward a plan, and depend on the public to comply with the new rules, leading to more collective action and less death [13]. Thus, it is imperative to have a better understanding of the role of trust in government in shaping the public’s reactions to a pandemic.

However, the current trust literature has two omissions. First, previous research has mainly focused on “trust in government” in general [14] or “trust in the federal government” in particular [15]. Yet, within the United States, there are multiple tiers of governance: the federal government operates at the national level and its powers are vested by the U.S. constitution in the Congress (the legislative branch), President (the executive branch), and the federal courts (the judicial branch). Importantly, however, under the 10th amendment to the U.S. constitution, any powers not specifically granted to the federal government are reserved to the states, which themselves have their own executive branch led by a governor, legislative branch, and judiciary. Thus, for individuals living within the U.S., the federal government represents just one of several foci for trust in government. Notably, the public may also have varying levels of trust toward their state government.

Much of the research in the trust field has focused solely on institutional trust in the federal government and largely overlooked whether trust in state government might make a difference to the attitudes, behavior, and well-being of the public. Distinguishing trust in federal government and trust in state government is especially important when facing COVID-19 in the U.S. because the federal government delegated primary responsibility for containing the virus to states, and the pandemic prompted different governmental responses from each state government [16]. For example, at the time of our data collection, the federal government was led by President Trump (a Republican) who delivered inconsistent messaging regarding the severity and expected course of the pandemic [17]. Moreover, approximately half of the states (24) were led by Democratic governors, whereas 26 were led by Republican governors. With vastly different strategies for dealing with the pandemic informed in part by their politics, some state governors chose not to take many restrictive actions or not to exercise their executive authority, believing that the public should instead voluntarily use their own discretion in taking preventative measures rather than have those be mandated by the state; in contrast, other state governors had more sweeping responses, closing restaurants, bars, and schools and implementing shutdowns and lengthy stay-at-home orders [18]. Because of the varying approaches across the federal and state governments, one may expect varying levels of trust in federal government and trust in state government.

The distinction between trust in federal government and trust in state government can be compared to the differences between trust in the supervisor and trust in the organization [19]. It is plausible that a person may trust the state but not the federal government. Likewise, a person may trust the federal government but not the state. They may trust both the state and federal government or not trust either state government or federal government. Thus, it is important to extend our current understanding of institutional trust to incorporate both foci—trust in federal government and trust in state government.

**Figure 1:**
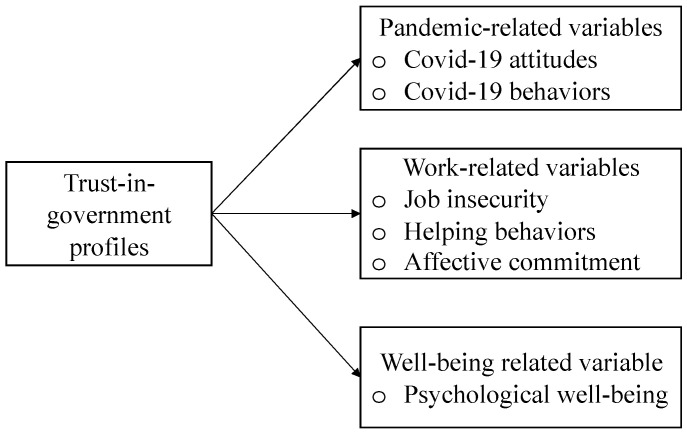
A conceptual model.

Second, to date, the most common way to examine institutional trust and trust in government is to ask the public to self-report the extent to which they trust the government or the institution of interest. That is, previous research has adopted a variable-centered strategy in which the independent relationships of trust in different foci with variables of interest are examined. However, such an approach does not account for the ways in which individuals may experience differing levels of trust in both federal government and state government. For example, in the face of COVID-19, some may have trust in both federal and state government, whereas others may primarily trust either the state government or the federal government. In other words, distinct profiles of institutional trust may exist. To investigate such a possibility, a person-centered approach is needed to understand the presence of distinct subpopulations of trustors that differentially experience trust in state government and trust in federal government. Given that institutional trust can be directed at multiple foci, the idea of a trust-in-government profile raises questions about how different profiles are differentially related to employees’ attitudes towards and behavioral compliance with COVID-19 prevention practices, job insecurity, helping behavior, affective commitment, and psychological well-being (see Figure 1 for a conceptual model).

In light of these unexplored areas of institutional trust, the current study makes three primary contributions to the extant literature. First, unlike the vast majority of previous studies that focus on only one aspect of institutional trust (e.g., [20]), we examine two foci of institutional trust (i.e., trust in federal government and trust in state government), thereby providing a more comprehensive understanding of trust in government. Second, unlike previous research that adopts a variable-centered approach (e.g., [21]), we use latent profile analysis (LPA [22]) to explore the potential existence of distinct latent trust-in-government profiles. LPA is designed to understand research questions that involve effects of qualitatively different configurations of variables that cannot easily be investigated by other methods. In doing so, we are able to identify how different subpopulations of employees characteristically experience distinct combinations of trust in federal government and trust in state government during the COVID-19 pandemic. For example, there might be qualitative differences (or shape differences) and quantitative differences (or level differences) between trust-in-government profiles. Third, we linked these profiles to various outcomes, which cast light on subpopulations under which trust in federal government and trust in state government might be more or less beneficial for employees’ attitudes towards and compliance with governmental policies, work-related variables, and well-being indicators. Because our study is conducted against the backdrop of COVID-19, we use people’s attitudes towards and behavior compliance with recommended prevention practices that aim to control the spread of COVID-19 to represent people’s attitudes towards and compliance with government health policies; to comprehensively represent people’s work experiences, we focus on employee job insecurity (a type of work stressor), workplace helping behaviors (a type of work behavior), and affective commitment (a type of work attitude). Finally, psychological well-being is used to evaluate people’s well-being. Thus, these three sets of outcomes allow us to paint a complete picture of the relevance of trust-in-government profiles that expands from attitudes towards and compliance with government health policies to people’s working and personal life. In what follows, we more fully delineate our proposed relationships.

### 1.1. A Person-Centered Approach to Trust in Government

The main aim of the current study is to identify distinct profiles of employees based on their levels of institutional trust towards two foci—state government and federal government. Given that this is the first study to apply a person-centered perspective to the study of trust in government and the first to do so with two foci of trust, we adopt an inductive approach to establish our trust-in-government profiles. We seek to assess whether distinct profiles exist. Thus, we are guided by the following general research question:

Research Question 1: Are there distinct profiles of trust in government?

We further explore three sets of outcomes of trust-in-government profiles. Below we discuss the rationale for examining each of these outcomes.

### 1.2. Trust-in-Government Profiles and COVID-19 Attitudes and Compliance

Trust in government positively relates to the extent to which individuals believe in and adopt recommended precautionary measures during a pandemic. For example, during the H1N1 pandemic, previous studies have found that trust in government is positively associated with vaccination intentions in the Netherlands [23], the acceptance of an Emergency Use Authorization drug for oneself in the U.S. [14], and hand hygiene in Hong Kong via greater understanding of H1N1 cause and H1N1 prevention self-efficacy [10]. In sum, studies conducted in various countries have confirmed that people who trust the government and authorities are willing to cooperate [24,25,26] and tend to display recommended behaviors more than those who do not trust the authorities [27]. Thus, we pursue the following research questions:

Research Question 2: Are trust-in-government profiles related to different levels of attitudes toward COVID-19 prevention practices?

Research Question 3: Are trust-in-government profiles related to different levels of behavioral compliance with CDC recommendations?

### 1.3. Trust-in-Government Profiles and Job Insecurity

Job insecurity can be defined as “perceived threat to the continuity and stability of employment as it is currently experienced” [28] (p. 1911). A number of factors at the individual, organizational, and society levels have been proposed to trigger job insecurity [29,30]. For example, Shoss identifies increased political uncertainty and instability as a predictor of job insecurity [28]. Because both the federal and state governments establish and enforce employment-related laws and regulations (e.g., restrictions on organizations’ ability to fire employees) and protect the rights of employees, the public can expect security in their employment if they believe that the government cares for their well-being and if they trust the government to make decisions that have significant impacts on their livelihood [31]. Moreover, Keele states that “trust is a reflection of government performance” [32] (p. 242). Thus, trust may be considered a barometer of how well government performs [33]. When trust in government is decreased as a result of poor economic growth, employees may also worry about the possibility of losing their employment. Relatedly, Anderson and Pontusson have revealed that governmental provision of social protection reduces employee job insecurity in 15 OECD countries [34]. Thus, we propose the following research question:

Research Question 4: Are trust-in-government profiles related to different levels of perceived job insecurity?

### 1.4. Trust-in-Government Profiles and Affective Commitment

Affective commitment to one’s organization can be defined as employees’ sense of belonging towards and identification with their employer, which increases their involvement in the organization’s activities, their willingness to pursue the organization’s goals, and their desire to stay with the organization [35,36]. Trust is crucial to maintain the exchange relationship between two parties [37]. In an organizational setting, trust in organization [38] and trust in management [39] are related to affective commitment.

Institutional trust is the belief that the government is honest and caring, has benevolent intentions towards the public, and makes an effort, in good faith, to react to the needs of the public [2]. Trust arises when government fulfills an explicit or implicit agreement with its citizens. When citizens trust their government, they have a generalized expectancy that their social environment can be relied upon [40]. Because individual attitudes and values (e.g., institutional trust) often color the way people interpret their environments and their relationships with this environment, it is possible that a high level of institutional trust may facilitate the development of high affective organizational commitment. Thus, we propose the following research question:

Research Question 5: Are trust-in-government profiles related to different levels of affective commitment?

### 1.5. Trust-in-Government Profiles and Helping Behavior

Defined as voluntarily helping others with, or the prevention of, work-related problems, helping behavior is an important type of organizational citizenship behavior [41]. Research has found that institutional trust facilitates social trust, or the belief that one can trust strangers [42]. More specifically, social trust indicates confidence in other people, believing that “people will manifest sensible and, when needed, reciprocally beneficial behavior in their interactions with others” [43] (p. 457). Building on the research findings that trust in the organizational setting, including interpersonal trust [44], trust in coworkers [45], and trust in supervisors [46], positively relates to helping behavior, trust in government may enhance helping behavior via social trust, where the helpers may expect reciprocally beneficial behavior from the receivers of help in the future. Moreover, people with high institutional trust also have confidence in the system and believe that the system will punish wrongdoers [47]. Thus, trust in government may prompt helping behavior because employees believe that the institution may induce others to reciprocate. Thus, we pursue the following question:

Research Question 6: Are trust-in-government profiles related to different levels of workplace helping behavior?

### 1.6. Trust-in-Government Profiles and Psychological Well-Being

Institutional factors impact well-being. Coleman in his seminal work proposes that trust is one of the three major forms of social capital [48]. Expanding upon the narrow conceptualization of trust in Coleman [49], trust in government can also be conceptualized as a type of social capital [50], thereby relating to higher levels of psychological well-being [51]. For example, trust in government may allow citizens to believe that they have sustained access to a wide range of resources (e.g., social security system [52]). Empirically, research has indicated that trust in national government is positively associated with people’s well-being based on data from China [53] and 15 European countries [54]. Moreover, institutional trust has been identified as a determinant of psychological well-being by empirical articles (e.g., [55]) and a systematic review [56]. Thus, we propose the following research question:

Research Question 7: Are trust-in-government profiles related to different levels of psychological well-being?

## 2. Method

### 2.1. Participants, Procedure, and Measures

To test our research propositions, we gathered two waves of anonymous survey data from 492 employed adults representing 48 states plus the District of Columbia in the United States using Amazon’s Mechanical Turk (MTurk), an online data collection crowdsourcing tool. Data collection was part of a larger ongoing study entitled, “Longitudinal Study of Work/Life Experiences During the COVID-19 Pandemic” (classified as exempt by the (author institution’s) IRB; protocol #18240). To facilitate the collection of high quality data and as recommended by Peer et al. [57], only “high reputation” MTurkers with a prior approval rating of 90%+ coupled with completion of at least 100 prior MTurk tasks were allowed to participate.

To reduce common method bias by providing temporal separation [58] between the data used to create respondent profiles and our dependent variables of interest, data on trust in federal government and trust in state government were collected in June of 2020 (Time 1), while all outcomes were collected in August of 2020 (Time 2). In exchange for their participation, respondents were paid $3.00 for the first wave, and a slightly higher amount ($3.50) for the second wave to incentivize continued participation. Of the 492 participants at Time 1, 427 returned to complete the Time-2 survey resulting in an 86% retention rate. Attrition analyses indicated no significant differences between Time-2 completers and Time-2 dropouts on the basis of sex, race, marital status, household income, age, work hours, or education level.

Table 1 presents the demographic information of participants.

Table 2 presents all measurement scales.

### 2.2. Statistical Analyses: Latent Profile Analysis (LPA)

All analyses were conducted with Mplus Version 8 [66] with robust maximum likelihood estimator (MLR). First, we ran confirmatory factor analyses to establish the validity of the two-factor trust construct. Second, the three-step approach was adopted to model auxiliary variables (i.e., outcomes [67]). In the first step, we conducted the LPA to determine the number of trust-in-government profiles that fit the data [67]. To select the best fitting profile solution, we considered both multiple fit values and content decision criteria. Specifically, we used a stepwise approach to determine the number of latent profiles, starting with a LPA with two profiles and successively adding profiles [68]. In each step, we examined seven fit information criteria, including log likelihood (LL), sample-sized-adjusted bootstrap likelihood ratio test (SABIC), bootstrap likelihood ratio test (BLRT), Lo-Mendell-Rubin likelihood ratio test (LMR), Akaike information criterion (AIC), Bayesian information criterion (BIC), and Entropy [69]. Although there are no cutoff scores for LPA fit statistics, the following rule of thumb is recommended: compared to other profile solutions, LL, AIC, BIC, and SABIC should be lower, while entropy should be larger; and LMR and BLRT should be statistically significant at *p* < 0.05. Second, based on the posterior distribution from the previous step [67], we obtained most likely class membership and determined the profile to which an individual is most likely to belong. Finally, we assessed the auxiliary variables in relation to the profile solution, considering the most likely class membership and classification error rate. We employed the Mplus DE3STEP command to test the outcomes of trust-in-government profiles [67]. Notably, the parameters that are fixed in Step 3 reflect membership probabilities, and are not directly associated with the means and variances of the focal indicators. As the auxiliary variables are not directly incorporated into profile estimation, this approach can account for individuals’ likelihood of being assigned to a single profile, while simultaneously avoiding shifts in latent profiles with the introduction of new variables in the subsequent steps [70], unless auxiliary variables were extremely non-normal [71]. This analysis also provides Wald chi-square statistics for examining whether there are statistically meaningful variations across all profiles on a given variable (overall), as well as across each pairing of profiles. Following guidelines by Spurk et al. [72], we applied MLR estimation and the full information maximum likelihood estimation (FIML) approach because of missing values caused by dropouts over time. To prevent local solutions, we set the number of random starts to 7000 and the final stage optimizations to 200 [73]. See Ram and Grimm for the order of decision steps [74].

## 3. Results

**Figure 2:**
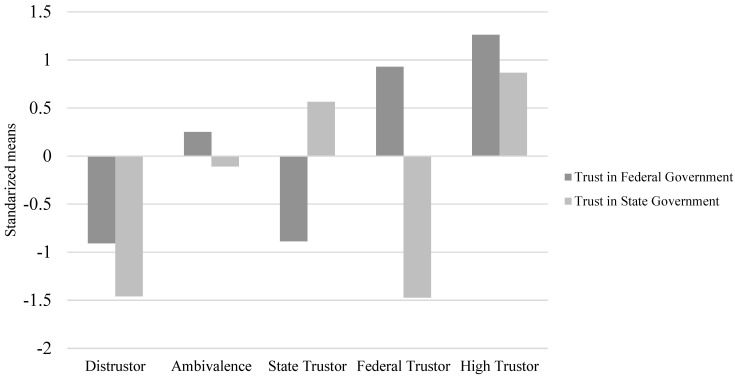
Standardized means in the indicators of trust in federal government and trust in state government for the five-profile solution.

Means, standard deviations, and correlations of variables are shown in Table 3. CFA results in Table 4 supported the two-factor trust model as the best fitting model. Table 5 shows the fit statistics for possible latent profile structures. We first considered the two-profiles solution, which showed the higher entropy value and a nonsignificant adjusted LMR-test when continuing to three profiles. However, the SABIC and AIC values kept descending with additional profiles. We chose the five-profile solution because it exhibited the highest value of Entropy, lower LL, AIC, BIC, and SABIC values, as well as significant LMR and BLRT values, in comparison to the two-, three-, and four-profile solutions. Although the six- and seven-profile solution had slightly lower LL, AIC, BIC, and SABIC values in comparison to the five-profile solution, the LMR or BLRT was nonsignificant. Finally, the five-profiles solution showed a number of different profiles of theoretical interest that are relatively different in content (see Figure 2).

Table 6 shows the mean of the trust indicators in each profile and counts of each profile for the sample. We labeled those in the most common profile as “high trustor” (*n* = 130, 26.5%), showing high levels of both trust in federal government (*M* = 5.486) and trust in state government (*M* = 5.774). The second group, termed as “state trustor” (*n* = 126, 25.7%), was characterized by low levels of trust in federal government (*M* = 1.777) but high levels of trust in state government (*M* = 5.278). We called the third group the “ambivalent” profile (*n* = 117, 23.9%) because it showed medium values for both foci of trust (*M*_trust federal_ = 3.741, *M*_trust state_ = 4.170). The fourth group was called “distruster” (*n* = 106, 21.6%) because they reported low values for both foci of trust (*M*_trust federal_ = 1.742; *M*_trust state_ = 1.948). We called the last group “federal trustor” (*n* = 11, 2.2%) because it featured high levels of trust in federal government (*M* = 4.912) but low levels of trust in state government (*M* = 1.928). These results revealed two qualitatively different profiles (state trustor and federal trustor) and three quantitatively different profiles (high trustor, ambivalent, and distruster). In response to Research Question 1, these results suggest that, quantitatively and qualitatively, different trust-in-government profiles exist in the sample.

Table 7 shows the outcomes of these trust-in-government profiles. State trustors reported the highest level of attitudes towards COVID-19 prevention strategies (*M* = 6.596), significantly higher than all other profiles, and the highest level of behavioral compliance with COVID-19 prevention strategies (*M* = 4.227), significantly higher than federal trustors, distrusters, and the ambivalent. High trustors reported the lowest levels of perceived job insecurity (*M* = 0.482), significantly lower than distrusters (*M* = 1.187), state trustors (*M* = 1.012), and ambivalent profiles (*M* = 1.017). High trustors also reported the highest levels of affective commitment (*M* = 5.369), significantly higher than all other profiles, as well as the highest levels of helping behavior (*M* = 5.916), significantly higher than distrusters (*M* = 5.105), state trustors (*M* = 5.607), and the ambivalent (*M* = 5.330). Federal trustors reported the highest level of psychological well-being (*M* = 5.015), significantly higher than distrusters (*M* = 4.061), while high trustors reported the second highest level of psychological well-being (*M* = 4.781), significantly higher than distrusters, state trustors (*M* = 4.374), and the ambivalent (*M* = 4.444). These results speak to the importance of Research Questions 2–6, indicating that different profiles of trust-in-government relate to different levels of attitudes towards and behavioral compliance with COVID-19 guidelines, job insecurity, affective commitment, helping behaviors, and psychological well-being.

## 4. Discussion

Adopting a person-centered approach to investigate trust-in-government profiles, we identified the existence of five distinct trustor profiles, or subpopulations: high trustors, federal trustors, state trustors, the ambivalent, and distrusters. In turn, these profiles were associated with different levels of attitudes towards and behavioral compliance with governmental policy relating to COVID-19, work-related variables including job insecurity, affective commitment, and helping behaviors, as well as psychological well-being.

### 4.1. Theoretical Implications

One major theoretical contribution of the current study is that we identify distinct latent trust-in-government profiles by simultaneously examining two foci of institutional trust (i.e., trust in federal government and trust in state government) and adopting a person-centered approach (i.e., LPA). Our results show five profiles that vary in their level (i.e., quantitative differences—distrusters, ambivalent, and high trustors) and shape (i.e., qualitative differences—federal trustors and state trustors). In other words, the three profiles of distrusters, the ambivalent, and high trustors indicate that trust in federal government and trust in state government can coexist within individuals at comparable levels. Moreover, the two profiles of federal trustors and state trustors indicate that individuals may only trust one institution but not the other, suggesting a negative relationship between trust in federal government and trust in state government among individuals in those profiles. In doing so, we show that trusting in one institution (e.g., one’s state) doesn’t necessarily mean trusting the other (i.e., the federal government). Although antecedents of these latent profiles were not explored in this study, this may be due to individuals whose trust in government is a function of political party. As such, if different political parties are in control at the state vs. federal level, this may manifest as distinct profiles of trust in state vs. federal government. This also implies that profile membership may shift over time as power shifts from one party to another in the state and/or federal governments. Unfortunately, because our data were collected prior to the November 2020 elections in the U.S., we could not test this proposition.

Our results also highlight how outcomes differ as a function of profile membership. Previous research found that when the public trusts their government, they perceive government health policies as legitimate and follow these based on positive expectations [75]. During the current pandemic, Dohle et al. found that trust in politics and trust in science are important predictors for the acceptance and adoption of preventative measures [76]. Interestingly, we find that state trustors report the highest level of attitudes towards COVID-19 prevention practices, which is significantly higher than all four other profiles, as well as the highest level of behavioral compliance with COVID-19 prevention strategies, which is significantly higher than three of the four other profiles (i.e., federal trustors, distrusters, ambivalence). In other words, state trustors, who only trust their local state government but not the federal government, were related to more positive attitudes toward and behavioral compliance with COVID-19 prevention measures compared to other profiles. As noted earlier, while this was an untested proposition, it may be that state-only trustors were primarily comprised of Democratic-leaning individuals living in Democratic-controlled states, which largely enacted more COVID-19 prevention policies compared to Republican-led states.

Theoretically speaking, given that trust-in-government profiles reveal varying attitudes toward and behavior compliance with recommendations to control the spread of COVID-19, this indicates the importance of incorporating both foci (i.e., trust in state government and the federal government) with a person-centered approach to understand institutional trust. LPA allows us to identify the unique subpopulation—state trustors who are most likely to have positive attitudes toward and behavioral compliance with CDC guidelines relating to stop the spread of COVID-19. Doing so responds to the call f researchers to understand factors that may account for variability in responses to COVID-19 prevention measures (cf. [77]).

On the other hand, high trustors report the highest levels of affective commitment and helping behaviors and the lowest level of job insecurity, which are significantly different from distrusters, state trustors, and those in the ambivalent profile. That is, trusting both federal and state government allows one to experience low job insecurity, report high affective commitment to the employer, and provide more helping behaviors towards others in the organization. Both state and federal government establish the rules and legal protections for employees. Thus, trusting both institutions, which influence one’s employment via regulations, decreases one’s perceived job insecurity. Moreover, research has suggested that institutional trust influences employees’ relationships with their employer and coworkers (cf. [78]). Thus, trusting both the state government and the federal government facilitates the development of affective commitment towards one’s organization. Finally, cross-national research has linked higher levels of trust in government to individual prosocial behavior [79]. Our research provides more nuanced evidence that trusting both state and federal government is the key to trigger helping behaviors in the organization, given that social trust is known to prompt employees to believe in and cooperate with others [80].

However, federal trustors experienced the highest level of psychological well-being, which is significantly higher than distrusters. High trustors report the second highest level of psychological well-being, which is significantly higher than distrusters, state trustors, and the ambivalent. These findings align with similar streams of research, which connect the government’s ability to provide a trustworthy environment with individual subjective well-being [81]. Recent research also indicates that: a person’s perceived efficacy of government actions is negatively associated with reported psychological distress [82]. Interestingly, our study reveals that, among all trust-in-government profiles, federal trustors experience the highest psychological well-being. It is plausible that the federal government, representing the highest level of power in the U.S., has most predictive power in terms of one’s well-being. Thus, to improve one’s well-being, it is important to make sure that the public have trust in the federal government.

### 4.2. Practical Implications

Our findings indicate that: (1) state trustors relate to the highest levels of attitudes toward and behavioral compliance with CDC recommended prevention guidelines; (2) high trustors relate to the highest levels of affective commitment and helping behavior and the lowest levels of job insecurity; and (3) federal trustors relate to the highest levels of psychological well-being. Consequently, distrusters and the ambivalent have low attitudes towards and behavioral compliance with prevention guidelines, low affective commitment, helping behavior, and psychological well-being, but high job insecurity. This indicates the importance of increasing people’s trust in state government and federal government. Doing so is particularly crucial given that trust has diminished in recent decades and has been found to decrease amid pandemic times [23]. Indeed, the Pew Research Center found that many U.S. adults believe that federal officials and news media withhold vital information from the public, and report challenges discerning what is true or false on social media platforms [83]. Thus, a major question remains as to how to rebuild institutional trust. Several studies suggest that institutional trust is related to government performance in the economy, citizen participation, and transparency [84,85]. With the COVID-19 pandemic as the backdrop, more recent research has found that governments perceived as well-organized, able to disseminate clear messages on COVID-19, and fair, are positively related to trust in government [79]. Together, federal and state officials can work to build trust through several avenues, such as practicing transparency, improving political leadership and government performance, participative problem solving, and clearly disseminating consistent messages on governmental policies.

Our findings also have implications beyond the outcomes examined. For example, although COVID-19 vaccination is now widely available, vaccine hesitancy is a roadblock to achieving a high vaccination rate. Research has suggested the vital role of institutional trust in vaccine uptake [14,23], where the public must trust the scientific community to produce safe and reliable vaccines and the government bodies to protect the public from harm from the uptake of vaccines.

At the organizational level, our findings indicate that management must be mindful of their workers’ trust toward the state and federal government because such trust, or lack thereof, has implications for employee job insecurity, affective commitment, and helping behaviors, in addition to attitudes towards and behavioral compliance with prevention practices and psychological well-being. For example, organizations may create a just organizational climate and have ethical leaders to facilitate employees’ trust in the organization, their leadership, and the department (e.g., [86]). Yet it may also be important to share information regarding the organization’s (hopefully, positive) interactions with state and federal regulators to build stronger trust in these institutions among employees.

### 4.3. Limitations and Future Directions

Although our study shows the vital role of trust-in-government profiles in employees’ perception, attitudes, and behaviors, there are some limitations. First, we collected all measures from the same source. Future studies should collect data from additional sources (e.g., supervisors or coworkers) on constructs such as helping behavior to minimize the common method bias [58]. Moreover, although we investigated how latent profiles of trust-in-government are related to various outcomes using a two-wave lagged dataset, the relationships between trust profiles and investigated outcomes are only theory driven. Like other correlational studies, our study does not allow us to draw causal conclusions because there might be other unexamined variables impacting the relationships among the variables of interest. Additionally, it is plausible that some of the examined outcomes (e.g., helping behavior) may be the antecedents of trust profiles [80]. For example, Sztompka has argued that “trust is the precondition for cooperation, and also the product of successful cooperation” [87] (p. 62). Thus, in the organizational setting, engaging in helping behaviors may facilitate institutional trust. Indeed, experimental studies and survey-based research have suggested that social trust is malleable, and its fluctuation largely stems from individuals’ interactions and experiences [88]. Future research may therefore consider bidirectional or cyclical relationships in the context of institutional trust.

Second, Spurk et al. recommended a minimum sample size of about 500 to achieve enough accuracy in identifying a correct number of latent profiles [72]. Our current sample size of 492 participants is slightly lower than this recommendation. Unfortunately, one of our profiles (i.e., the federal trustor) has a very small number of cases (*n* = 11). We kept this profile because it is theoretically meaningful (in that it qualitatively differs from the state trustor profile), and accounts for 2.3% of the total sample (which exceeds the rule of thumb of 1% [89]). Although this small profile size might be an artifact of a smaller-than-ideal overall sample size, including this profile might result in lower power, lower precision relative to other larger profiles, and less parsimony [89]. Thus, readers should be cautious when interpreting the results relating to the federal trustor profile, and future research should replicate our findings with a larger sample size.

Third, we used a convenience sample, which raises the issue of generalizability of our findings. For example, our sample was highly educated (66% of respondents had a college degree or higher compared to 32% in the general population) and had a higher percentage of males (61% compared to 49%) [90]. However, our respondents showed similar characteristics with the U.S. population regarding racial diversity (70% White compared to 76% in the population) and household income (median income ranging from $60,000–69,999 compared to $63,000 in the population) [90]. Cautions should be made when generalizing our findings to the entire U.S. population. Future research should therefore replicate our findings with a more representative sample.

As the first attempt, we did not examine the underlying mechanisms linking trust-in-government profiles and outcomes of interest. Future research may continue this line of research and examine the plausible mediators that may explain the associations between trust-in-government profiles and outcomes of interest (e.g., social trust [91]). Relatedly, because of our interest in the consequences of trust-in-government profiles, we did not examine the antecedents of trust-in-government profiles. As indicated earlier, preferences for the political party in power may in fact predict trust-in-government profiles. Similarly, it would be important to determine whether membership in the trust profiles shifts as elections take place and the political party in power at the state and/or federal-level also shifts. For example, following the November 2020 election, control of the White House and the Senate both shifted from Republican control to Democratic control. Therefore, it is possible that individuals who were initially members of a profile with higher trust in the federal government may have shifted to one of the profiles with low trust in the federal government following that election. Likewise, one might observe that individuals who were initially members of a profile with lower trust in the federal government may have shifted to one of the profiles with higher trust in the federal government following the 2020 election. Additionally, other major contextual events may also influence the potential fluctuation of trust profiles. For example, the Coronavirus Aid, Relief, and Economic Security (CARES) Act made by Congress in late March 2020 is likely to be relevant to changes in trust profiles. This act includes an aid package, designed in part to assist households in the COVID-19 derived economic crisis, with a stimulus check program offering up to $1200 for each eligible adult and $500 per child to eligible families [92]. Using a sample based in Detroit, Michigan, Wileden and Rodems found that those who did not receive a stimulus check payment reported significantly lower levels of trust in the government than those who had [93]. Together, future research may investigate the role of political party preferences and influencing political events (e.g., changes in parties and political power) in impacting trust-in-government profile membership and their correlates.

Indeed, the ever-changing landscape of the COVID-19 pandemic requires our adaptation to a new way of life, such as working from home, remote learning, contact tracing, social distancing, and wearing face masks when going out. To cope with the pandemic, state and federal government also constantly update their guidelines (e.g., vaccination pass and vaccine booster) and protection framework (e.g., from Alter Levels to Traffic Lights in New Zealand). Therefore, future research may consider these changing factors when examining institutional trust (e.g., how the continually evolving governmental guidelines themselves may erode or enhance trust among citizens).

## 5. Conclusions

“Trust is one of the most important synthetic forces within society” [94] (p. 326). Given the important role of institutional trust (e.g., [9]), a greater understanding of trust in government is critical. Through a person-centered lens, we use the LPA approach to examine trust-in-government profiles with two foci—trust in state government and trust in federal government—and their linkages with COVID-19-related variables (i.e., employees’ attitudes toward and behavioral compliance with CDC guidelines) as well as work-related outcomes (i.e., job insecurity, affective commitment, workplace helping behavior) and a well-being indicator (i.e., psychological well-being). This study reveals five distinct trust profiles (i.e., high trustors, state trustors, the ambivalent, distrusters, and federal trustors). That is, the three profiles of distrusters, the ambivalent, and high trustors reveal that individuals may have comparable levels of trust in federal government and trust in state government, while the two profiles of federal trustors and state trustors reveal that individuals may only trust one institution but not the other. Interestingly, our results also demonstrate that each profile has differing relationships with the outcomes of interest, where state trustors have the highest levels of positive attitudes towards and behavioral compliance with CDC guidelines relating to control the pandemic, high trustors have the highest levels of affective commitment and helping behaviors but the lowest levels of job insecurity, and federal trustors have the highest levels of psychological well-being. Our results show the benefits of adopting a person-centered approach to better explicate institutional trust.

## Figures and Tables

**Table 1:** Demographic information of participants.

Demographic Categories	Percentage
Gender	
Male	60.9
Female	39.1
Race	
African American or Black	11.5
American Indian or Native American	0.6
Asian or Pacific Islander	7.4
Anglo/White	69.8
Hispanic or Latino/a	9.3
Other	1.3
Education	
Less than a high school diploma	0.3
High school diploma or GED	8.7
High school diploma plus some technical training or apprenticeship	1.0
Some college	22.8
Graduate from college (BA/BS or other Bachelor’s degree)	49.8
Some graduate school	2.4
Graduate or professional degree	15.0
Marital status	
Married or living with a partner	55.7
Separated	1.4
Divorced	5.7
Widowed	0.5
Never married	36.6
Income	
Less than $10,000	1.9
$10,000 to $19,999	6.6
$20,000 to $29,999	8.6
$30,000 to $39,999	12.8
$40,000 to $49,999	12.5
$50,000 to $59,999	11.6
$60,000 to $69,999	11.6
$70,000 to $79,999	10.1
$80,000 to $89,999	5.0
$90,000 to $99,999	4.8
$100,000 to $149,999	10.2
$150,000 or more	4.4
Industrial sectors	
Accommodation or food services	4.9
Administration and Support Services	4.6
Agriculture, Forestry, Fishing, and Hunting	0.8
Arts, entertainment or recreation	3.1
Construction	3.5
Educational Services	8.1
Finance or Insurance	11.7
Health Care or Social Assistance	9.1
Information	8.1
Management of companies or enterprises	2.8
Manufacturing	8.2
Mining, Quarrying, and Oil and Gas Extraction	0.1
Other Services	1.9
Professional, Scientific or Technical Services	13.7
Real Estate, Rental and Leasing	1.2
Retail Trade	11.3
Self-Employed	0.9
Transportation or Warehousing	3.5
Utilities	0.6
Other	2.1

**Table 2:** Measurement scales used in this study.

Variable	Definition	Items & Origin	Response Option	Reliability
Trust in federal government (Time 1)	Beliefs about the degree to which the federal government are honest, care for the public, have benevolent and caring intentions towards the public, and make a good faith effort to react to the needs and concerns of the public [2].	Read each statement and indicate the extent to which you agree or disagree with each [2]. (1) I trust the leaders of the federal government to make decisions that are good for everyone in the U.S. (2) People’s basic rights are well protected by the federal government (3) The federal government cares about the well-being of everyone in the U.S. (4) The federal government is honest (5) The federal government considers the views of the people in the U.S. (6) The federal government takes account of the needs and concerns of the people in the U.S. (7) The federal government gives honest explanations for their actions to the people in the U.S.	1–7 strongly disagree to strongly agree	0.96
Trust in state government (Time 1)	Beliefs about the degree to which the state government are honest, care for the public, have benevolent and caring intentions towards the public, and make a good faith effort to react to the needs and concerns of the public [2].	Read each statement and indicate the extent to which you agree or disagree with each [2]. (1) I trust the leaders of the state government to make decisions that are good for everyone in the state (2) People’s basic rights are well protected by the state government (3) The state government cares about the well-being of everyone in the state (4) The state government is honest (5) The state government considers the views of the people in the state (6) The state government takes account of the needs and concerns of the people in the state (7) The state government gives honest explanations for their actions to the people in the state	1–7 strongly disagree to strongly agree	0.97
Attitudes toward COVID-19 prevention guidelines (Time 2)	The extent to which individuals have positive attitudes toward the recommended COVID-19 prevention guidelines [59]	Please indicate your thoughts regarding physical/social distancing and hygiene recommendations from the CDC [59]. (1) It is important to maintain a distance of at least 6 ft. from others when out in public or at work. (2) Staying at home except to conduct essential tasks (e.g., grocery shopping, medical appointments) is an effective way of stopping the spread of COVID-19. (3) Disinfecting frequently used items and surfaces is beneficial to prevent spreading the virus. (4) Frequently washing hands for a minimum of 20 s can reduce the spread of the virus. (5) Avoiding touching my face will help protect me from COVID-19. (6) When coughing or sneezing, people should aim inside their elbow or into a tissue. (7) The economic cost of social distancing measures is worth the price to protect public health. (8) Wearing a mask (e.g., N95 respirator masks, medical masks, or fabric masks) while out in public can reduce my chances of potentially spreading COVID-19 to others.	1–7 strongly disagree to strongly agree	0.90
Behavioral compliance with COVID-19 prevention guidelines (Time 2)	The extent to which individuals comply with the recommended COVID-19 prevention guidelines [60]	Please indicate how often you currently engage in the following behaviors to prevent spreading the coronavirus [60]. (1) I maintain a distance of at least 6 ft. from others when out in public or at work; (2) I stay at home except to conduct essential tasks (e.g., grocery shopping, medical appointments). (3) I disinfect frequently used items and surfaces. (4) I frequently wash hands for a minimum of 20 s. (5) I avoid touching my face. (6) When coughing or sneezing, I aim inside my elbow or into a tissue. (7) I wear a mask (e.g., N95 respirator masks, medical masks, or fabric masks) when I am out in public.	1–5 Never to always	0.84
Perceived job insecurity (Time 2)	“perceived threat to the continuity and stability of employment as it is currently experienced” ([28], p. 1911)	What is your FUTURE EMPLOYMENT like in your organization? [61] My future employment is: (1) Sure (2) Unpredictable (3) Up in the air (4) Stable (5) Questionable (6) Unknown (7) My job is almost guaranteed (8) Can depend on being here (9) Certain	No Don’t know Yes	0.97
Affective commitment (Time 2)	Employees’ sense of belonging towards and identification with their employer that increases their involvement in the organization’s activities, their willingness to pursue the organization’s goals, and their desire to stay with the organization [35]	The following statements are about how you feel about your organization. Please read each statement carefully and indicate to what extent you agree that each of the following statements [62]. (1) I would be very happy to spend the rest of my career with this organization. (2) I really feel as if this organization’s problems are my own. (3) I do not feel a strong sense of “belonging” to my organization. (4) I do not feel “emotionally attached” to this organization. (5) I do not feel like “part of the family” at my organization. (6) This organization has a great deal of personal meaning for me.	1–7 strongly disagree to strongly agree	0.94
Helping behaviors (Time 2)	Voluntarily helping others with, or the prevention of, work-related problems [41]	Read each statement and indicate the extent to which you agree or disagree with each [63]. (1) I help others who have been absent. (2) I help others who have heavy workloads. (3) I take time to listen to co-workers’ problems and worries. (4) I go out of my way to help new employees.	1–7 strongly disagree to strongly agree	0.88
Psychological well-being (Time 2)	The extent to which individuals experience positive emotions [64].	During the past month… [65] (1) How often have you been a very nervous person? (2) How often have you felt calm and peaceful? (3) How often have you felt downhearted and blue? (4) How often have you felt so down in the dumps that nothing could cheer you up? (5) How often were you a happy person?	1–6 none of the time to all of the time	0.90

**Table 3:** Means, standard deviations, and correlations of all variables.

	N	M	SD	1	2	3	4	5	6	7	8
1. Trust in federal government	492	3.31	1.60	0.96							
2. Trust in state government	492	4.26	1.51	0.45 **	0.97						
3. COVID-19 attitudes	422	6.18	1.00	−0.07	0.19 **	0.90					
4. COVID-19 behaviors	410	3.99	0.69	−0.01	0.20 **	0.73 **	0.84				
5. Job insecurity	399	0.91	1.18	−0.20 **	−0.16 **	0.00	−0.04	0.97			
6. Helping behavior	386	5.50	1.13	0.19 **	0.27 **	0.19 **	0.32 **	−0.24 **	0.88		
7. Affective commitment	399	4.55	1.69	0.28 **	0.28 **	0.07	0.25 **	−0.45 **	0.46 **	0.94	
8. Psychological well-being	422	4.44	1.20	0.18 **	0.17 **	0.10 *	0.15 **	−0.36 **	0.30 **	0.40 **	0.90

Note. Reliabilities are along the diagonal. * *p* < 0.05; ** *p* < 0.01. Trust was measured at Time 1. All other variables were assessed at Time 2.

**Table 4:** Results of Confirmatory Factor Analyses of Institutional Trust Construct.

Model	χ^2^	*df*	CFI	TLI	RMSEA	SRMR
One-factor	2522.25	77	0.535	0.450	0.255	0.255
Two-factor	372.57	76	0.944	0.932	0.089	0.024

Note. CFI = comparative fit index; TLI = Tucker-Lewis index; RMSEA = Root mean square error of approximation; SRMR = standardized root-mean-square residual.

**Table 5:** Fit Indices of Profile Indicators.

No. of Profiles	LL	FP	SABIC	BLRT (*p*)	LMR (*p*)	AIC	BIC	Entropy
2	−1301.11	7	2623.35	0.0000	0.0000	2616.21	2645.57	0.840
3	−1258.43	10	2547.05	0.1371	0.1260	2536.85	2578.79	0.763
4	−1205.60	13	2450.46	0.0129	0.0150	2437.19	2491.72	0.833
5	−1185.18	16	2418.68	0.0057	0.0071	2402.35	2469.46	0.862
6	−1165.85	19	2389.08	0.1637	0.1741	2369.69	2449.38	0.841
7	−1152.87	22	2372.20	0.0785	0.0856	2349.75	2442.02	0.831

Note. LL = log-likelihood; FP = free parameters; SABIC = Sample-size-adjusted Bayesian information criteria; BLRT (*p*) = *p*-Value for the bootstrapped likelihood ratio test. LMR (*p*) = *p*-Value for the adjusted Lo-Mendell-Rubin-test; AIC = Akaike information criteria; BIC = Bayesian information criteria.

**Table 6:** Profile Counts and Trust Means.

Profile	N	% of Sample	Trust in Federal Government	Trust in State Government
High Trustor	130	26.5%	5.486	5.774
State Trustor	126	25.7%	1.777	5.278
The ambivalent	117	23.9%	3.741	4.170
Distrustor	106	21.6%	1.742	1.948
Federal Trustor	11	2.3%	4.912	1.928

**Table 7:** Three-Step Results for Outcomes of Trust-in-government Profiles.

	Federal Trust (a)	Distrust (b)	High Trust (c)	State Trust (d)	Ambivalent (e)	Chi Square
COVID-19 Attitudes	5.892 _d_	6.068 _d_	6.252 _d,e_	6.596 _a,b,c,e_	5.849 _c,d_	42.02 ***
COVID-19 Compliance	3.750 _c,d_	3.914 _d_	4.105 _a,e_	4.227 _a,b,e_	3.743 _c,e_	21.93 ***
Perceived job insecurity	0.877	1.187 _c_	0.482 _b,d,e_	1.012 _c_	1.017 _c_	26.71 **
Affective commitment	3.714 _c_	3.804 _c,d,e_	5.369 _a,b,d,e_	4.572 _b,c_	4.408 _b,c_	55.438 ***
Helping behavior	4.819	5.105 _c,d_	5.916 _b,d,e_	5.607 _b,c_	5.330 _c_	36.24 ***
Psychological well-being	5.015 _b_	4.061 _a,c_	4.781 _b,d,e_	4.374 _c_	4.444 _c_	19.40 **

Note. The values for attitudes, compliance, job insecurity, affective commitment, helping, and psychological well-being for each profile are means. ** *p*< 0.01, *** *p* < 0.001. Each subscript indicates that there is a significant difference between the target profile and the other profile(s).

## Data Availability

The data presented in this study are available on request from the corresponding author. The data are not publicly available due to privacy concerns.
